# Supplementary material for: The causal relationship between the human gut microbiota and pyogenic arthritis: a Mendelian randomization study
Source: Front Cell Infect Microbiol. 2024 Nov 26;14:1452480. doi: 10.3389/fcimb.2024.1452480 (PMC11629706; doi:10.3389/fcimb.2024.1452480)
Supplement: Supplementary file 1 [file DataSheet1.zip › mendelian test/ebi-a-GCST90027642.csv_leave_one_out.pdf]

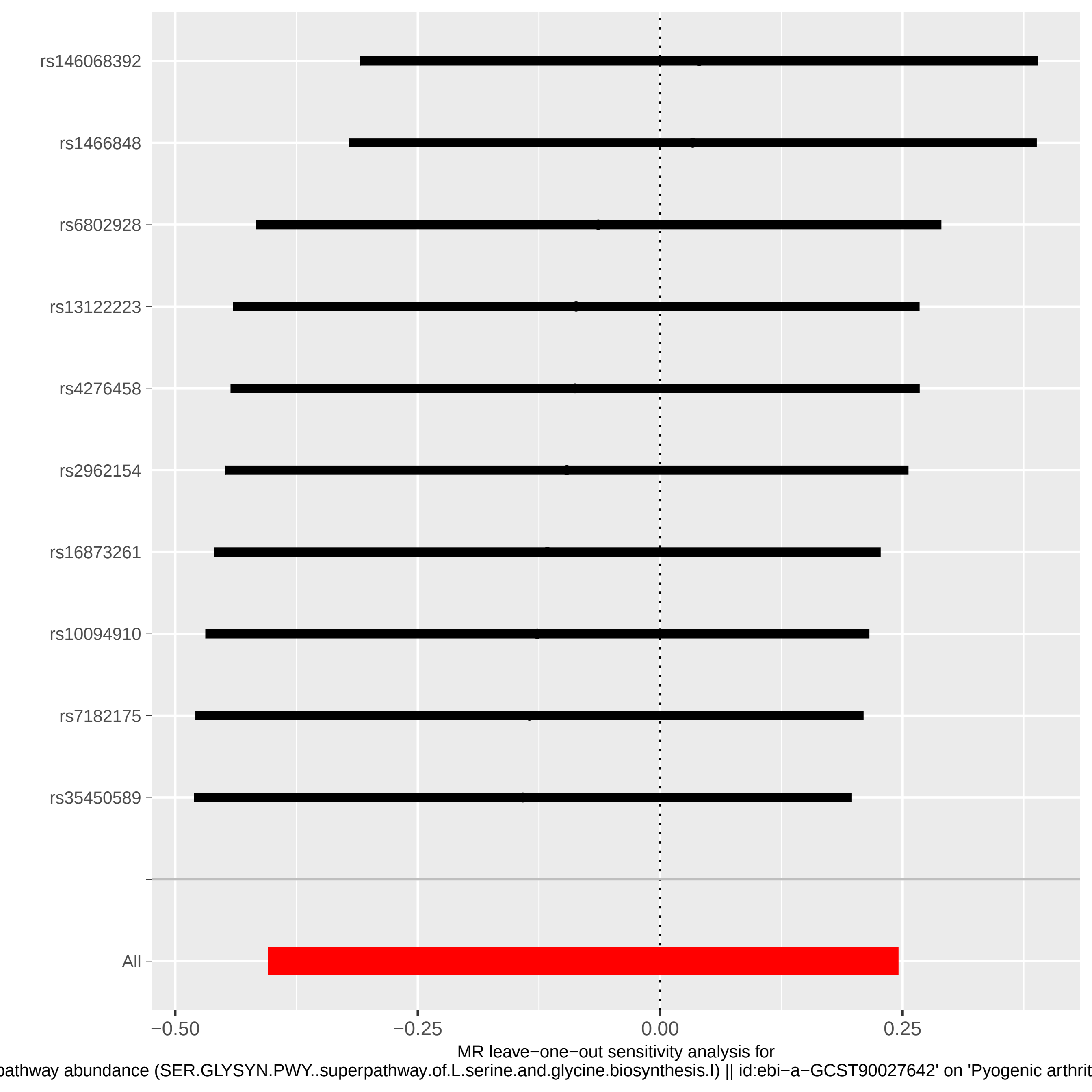

rs146068392
rs1466848
rs6802928
rs13122223
rs4276458
rs2962154
rs16873261
rs10094910
rs7182175
rs35450589
All
−0.50
−0.25
0.00
0.25
MR leave−one−out sensitivity analysis for
pathway abundance (SER.GLYSYN.PWY..superpathway.of.L.serine.and.glycine.biosynthesis.I) || id:ebi−a−GCST90027642' on 'Pyogenic arthrit
